# Adult Male Rats Show Resilience to Adolescent Bisphenol A Effects on Hormonal and Behavioral Responses While Co-Exposure With Hop Extracts Supports Synergistic Actions

**DOI:** 10.3389/ftox.2021.639820

**Published:** 2021-05-14

**Authors:** Alexandre Morin, Lise Van de Beeck, Emmanuelle Person, Helene Plamondon

**Affiliations:** Behavioural Neuroscience Group, School of Psychology, University of Ottawa, Ottawa, ON, Canada

**Keywords:** bisphenol A, hop extracts, adolescence, corticosterone, testosterone, anxiety, depression, sociability

## Abstract

The adolescence period, marked by sexual and brain maturation, has shown sensitivity to various environmental disruptors. Exposure to the xenoestrogen bisphenol A (BPA) is known to alter physiological and behavioral responses although its role at this critical period remains largely unknown. Recent research further suggests biochemical and genomic effects of BPA to be mitigated by various natural compounds, while effects on behavior have not been examined. This study aimed to characterize (1) the effects of dietary BPA during adolescence on endogenous corticosterone (CORT) secretion, emotional behavior, and testosterone (T) in adulthood, and (2) the impact of combined exposure to BPA with hop extracts (Hop), a phytoestrogen with anxiolytic properties. To do so, four groups of male Wistar rats [postnatal day (PND) 28] were administered corn oil (control), BPA (40 mg/kg), hops (40 mg/kg), or BPA-hops by oral gavage for 21 days (PND 28–48). Blood droplets collected on PND 28, 48, and 71 served to measure CORT and T changes. As adults, rats were tested in the elevated plus maze (EPM), the social interaction test, and the forced swim test. Our findings demonstrated elevated anxiety and a trend toward depressive-like behaviors in BPA- compared to hops-exposed rats. However, BPA intake had no impact on basal CORT levels, or adulthood T secretion and sociability. Of note, BPA's anxiogenic effect manifested through decreased EPM open arm entries was abolished by hops co-supplementation. Together, our observations suggest the adolescence period to be less sensitive to deleterious effects of BPA than what has been reported upon gestational and perinatal exposure.

## Introduction

Bisphenol A (BPA) is a pollutant used in the manufacturing of polycarbonate plastics and resins used in many food containers, toys, and dental equipment, whose presence is associated with food and water contamination. Dietary BPA exposure has been linked to long-term adverse health effects, including endocrine dysregulation (Fujiwara et al., 2016). In this context, upregulation of basal and stress-induced corticosterone (CORT) levels has been observed following gestational and lactational low-dose BPA exposure (40 μg/kg/day) in male rats (Panagiotidou et al., 2014) while perinatal and adult low- and high-dose exposure increased anxiety and depressive-like behavior (Matsuda et al., 2012; Chen et al., 2014; Xu et al., 2015). However, other studies have reported reduced anxiety or no effects following perinatal BPA exposure (Farabollini et al., 1999; Jones and Watson, 2012; Wise et al., 2019). Growing evidence further supports BPA-induced dysregulation of the hypothalamic-pituitary-gonadal (HPG) axis, ultimately affecting estrogen (E) and testosterone (T) secretion. Interestingly, studies indicate BPA supplementation timing to be determinant, with gestational and mid-adolescent/young-adulthood exposure decreasing rodents' T secretion (0.5–50 μg/ml, Takao et al., 1999; 2 μg/kg, Chen et al., 2014; 20 μg/kg, Hong et al., 2016), while perinatal BPA administration increased or had no influence on T levels (Fernández et al., 2010; Bowman et al., 2014). Possibly associated with altered gonadal hormone secretion, studies have supported gestational and transgenerational BPA exposure to hinder juvenile sociability (Rebuli et al., 2015) and social recognition (Wolstenholme et al., 2019). To date, most research on BPA has been conducted in the perinatal setting, leaving a paucity of research addressing exposure to pharmacological doses during adolescence. Nonetheless, this stage is important to examine considering the likelihood for BPA's effects to differ throughout the lifespan, and that adolescence plays a critical role in socioemotional and endocrine maturation.

Hop extracts (Hop) are polyphenolic compounds extracted from the female inflorescence of the *Humulus lupulus L*. (*Cannabaceae*) plant, which are often used as a bittering and conservation agent in the brewing industry. As members of the lupulin bioactive family, hops are composed of many bioactive molecules that impact psychological and physiological health. These molecules include soft resins (α and β bitter acids), hard resins [xanthohumol (XN) polyphenol and metabolites 6- and 8-prenylnaringenin (6- and 8-PN, respectively)], as well as essential oils (hydrocarbons and oxygen fractions) (Zanoli and Zavatti, 2008; Bertelli et al., 2018; Lin et al., 2019). Xanthohumol (XN), a prenylated chalcone constituting the major part of hop polyphenols, has recorded strong antioxidant properties in addition to anti-obesity, antiplatelet, and chemoprotective actions (Zanoli and Zavatti, 2008; Lin et al., 2019), while 6- and 8-PN demonstrate phytoestrogenic properties, with the latter showing the strongest influence (Zanoli and Zavatti, 2008). A growing number of recent reports have supported beneficial health effects of hop extracts (Karabin et al., 2015), including anxiolytic activity in humans (Kyrou et al., 2017) and animals (Zanoli and Zavatti, 2008). Studies have shown hop extracts administration to reduce behavioral despair in the forced swim test (FST) in rodents (Zanoli and Zavatti, 2008; Preedy, 2011), as well as ameliorate depression scores in young adults (Kyrou et al., 2017). While hops supplementation induces pro-estrogenic effects associated with increased E expression (Kazeruni et al., 2014), effects on testosterone (T) secretion have been contradictory (Kazeruni et al., 2014; Karbalaei et al., 2019). However, effects of hops exposure on sociability during development or adulthood remain largely unknown.

Notably, research indicates common mechanisms by which BPA and hop extracts could influence hormonal and socio-emotional responses. To date, research has examined isolated effects of dietary BPA supplementation, an approach that facilitates interpretation of underlying mechanisms. However, in view of the complex interactions at play, it may lead to misunderstandings about a compound's importance relative to other environmental factors. Indeed, BPA co-exposure with a series of dietary and naturally occurring compounds has strongly affected its related toxic and genomic effects (Sonavane and Gassman, 2019). In this context, existing studies support BPA and hop extracts to mediate hormonal and behavioral responses using similar physiological pathways (Poimenova et al., 2010; Kazeruni et al., 2014; Chen et al., 2015; Donoso et al., 2020). To date, the effects of adolescent BPA-hops co-exposure have not been studied. This is intriguing considering the heightened brain plasticity and vulnerability to a myriad of exogenous influences observed at this period (Kolb and Gibb, 2011) as well as the critical impact of stress exposure experienced during this window on delayed adulthood stress response and cognition (Chaby et al., 2013; Hodges et al., 2018). To our knowledge, the study by Gao et al. (2020) is the only one assessing selective, long-lasting effects of daily BPA exposure covering exclusively the adolescence period [18-day administration from postnatal day (PND) 28]. Their findings show BPA administration to be associated with reduced pubertal social play, while lower BPA dosages (0.04 mg/kg) reduced social interaction with female but not male mice. Other studies have used shorter BPA supplementation regimens that do not cover the entire adolescent period (Bowman et al., 2014) or longer regimens that carry over into adulthood (Takao et al., 1999; Xu et al., 2012).

This study aimed to investigate the impact of 21-day dietary exposure to BPA and hops during adolescence on CORT secretion profile, and characterize delayed effects on anxiety, sociability, and T secretion in adulthood. A second objective was to determine if hop extracts can mitigate the immediate and delayed behavioral and endocrine effects of adolescent BPA exposure. To date, the majority of studies have assessed BPA effects associated with gestational and early postnatal exposure while effects of hops have only been examined in adult rodents. Defining isolated and combined effects of hops and BPA during the pivotal adolescence period will support a better understanding of the abiding effects of environmental factors on adult biobehavioral responses.

## Materials and Methods

### Animals

**Figure 1:**
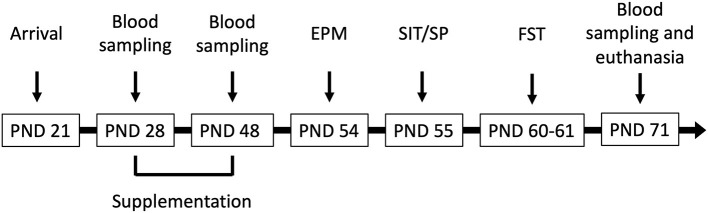
Male Wistar rats (*N* = 41) arrived at the facility on postnatal day (PND) 21 and were supplemented corn oil (*n* = 10; control), bisphenol A (BPA; 40 mg/kg; *n* = 10), hop extracts (40 mg/kg; *n* = 11), or a BPA-Hop mix (*n* = 10) from PND 28–48. Afterwards, rats were tested in the elevated plus maze (EPM), the social interaction/preference tests (SIT/SP), and the forced swim test on PND 54, 55, and 60–61, respectively. Blood samples were taken on PND 28, 48, and 71. Rats were euthanized on PND 71 following the last blood collection.

Male Wistar rats (*N* = 41) aged PND 21 (weighing 50–60 g upon arrival) were obtained from Charles River Laboratories (Rochefort, Québec, Canada) and were housed two/three per cage with daily gentle handling to minimize stress. They were maintained on a 12 h light/dark cycle (lights on at 7 A.M.) with free access to water and standard rat chow (2018 Teklad Global 18% Protein Rodent Diet, Envigo, USA). This diet does not contain alfalfa, therefore reducing the amount of natural phytoestrogens. Room temperature was set at 21–23°C with 60% relative humidity. Cages and water bottles were composed of polycarbonate. BPA levels from polycarbonate bottles yield a potential of ~0.04 to 0.08 μg/kg/day for an ~50 ml daily water consumption (Honeycutt et al., 2017). All experimental manipulations were conducted between 8:30 A.M. and 7 P.M. and a timeline can be found in Figure 1. All procedures were carried out in accordance with the Canadian Council on Animal Care and were approved by the University of Ottawa Animal Care Committee. Experimentation complied with the ARRIVE guidelines and was in accordance with the National Institutes of Health guide for the care and use of laboratory animals (NIH Publication's No. 8023, revised 1978).

### Drug Preparation

All solutions were stored at 4°C and left to sit at room temperature (21–23°C) for at least 30 min prior to gavage sessions. BPA (2 g) was mixed in corn oil (5 ml) to obtain a 40 mg/ml solution. Hop extracts (2 g) highly concentrated in α and β acids (68.7%; Table 1) were obtained from Yakima Valley Hops (Yakima, Washington, USA) and mixed in corn oil (5 ml) for 1 h at room temperature to obtain a 40 mg/ml concentration. To optimize dissolution, the preparation was mixed overnight. BPA-Hop: BPA (2 g) and hop extracts (2 g) were mixed overnight in corn oil (5 ml) to obtain a solution containing 40 mg/ml of each compound. Solutions were renewed every week to prevent oxidation.

**Table 1:** Composition of hop extract.

**Constituents**	**Percentage of extract (%)**
**α acids**	
Humulones	37.5
Cohumulones	15.5
**β acids**	
Lupulones	7.2
Colupulones	8.5
**Aroma**	31.3

*Alpha and beta acid constituents in the supplemented hop extracts as determined by high-performance liquid chromatography (as per supplier data sheet)*.

### Drug Administration

Animals had a 1-week habituation to the animal facility with only daily gentle handling prior to initiation of oral gavage. They were orally administered 1–2 droplets of condensed milk through the syringe in the last 3 days to facilitate later gavage regimen. Daily drug administration (2 ml/kg) extended from PND 28 to 48 (between 8 and 11 A.M.), covering the juvenile and adolescent period. Animals were weighed daily prior to gavage in order to prepare weight-appropriate solutions and gavage was performed by inserting a 20-gauge gavage needle into the esophagus to reach the stomach where the substance was slowly released. Using doses under 10 ml/kg, gavage administration has been shown to induce minimal stress 1 h after the procedure (Brown et al., 2000), and to be less stressful than restricted feeding periods in adolescent rats (Raymond et al., 2020). Administration volume was 2 ml/kg body weight. BPA and hops dosages were selected from prior studies (*BPA:* Richter et al., 2007; Jašarević et al., 2011; *hops:* Schiller et al., 2006; Karabin et al., 2015; Karbalaei et al., 2019).

Animals were randomly divided into one control and three experimental exposure groups: *Control* (*n* = 10): corn oil; *Hop* (*n* = 11): hop extracts (40 mg/kg); *BPA* (*n* = 10): BPA (40 mg/kg); *BPA-Hop* (*n* = 10): BPA (40 mg/kg) + hop extracts (40 mg/kg).

### Blood Sampling

Blood samples were collected at three distinct intervals (PND 28, 48, and 71). A small needle prick on the lateral tail vein allowed collection of two blood drops on Whatman blood stain cards (Sigma-Aldrich, Canada). This method has been used repeatedly and has been shown to reliably measure CORT values with minimal amounts of blood (Milot et al., 2012). All samples were collected between 8 and 10 A.M. On the days that coincided with drug administration, samples were collected prior to gavage. All samples were allowed to dry overnight at room temperature before being stored in the −80°C freezer until they were eluted from the cards for further analysis by enzyme-linked immunosorbent assay (ELISA).

### Corticosterone and Testosterone Immunoassays

An ELISA kit (Corticosterone EIA kit, AD1-901-097, ENZO Life Sciences) was used to assess duplicates of blood droplets for circulatory CORT levels as previously demonstrated (Raymond et al., 2020). Manufacturer instructions were followed for standards and samples preparation. After removal from the −80°C freezer, Whatman blood stain cards were left at room temperature (RT) for 30 min to thaw. Blood samples were punched in circular (*D* = 3 mm) shapes using a Gem Hole Punch (McGill Inc., Marengo, IL) from blood sample cards and were left in parafilm-sealed glass tubes with 280 μl of assay buffer on a Belly Dancer^®^ (Structure Probe Inc., West Chester, PA) for 24 h at RT. Then, to prevent non-specific CORT-protein binding, 5.5 μl of steroid displacement reagent (SDR) was added to 214.5 μl of each sample after incubation. Assay buffer was used to set sample dilution of 1:5 before scanning by PowerWave™ XS2 Microplate Spectrophotometer (BioTek, Winooski, VT) to determine CORT absorbance. Four-parameter equation derived from standard curve values was used to assess concentration. Assay analytic range was 32–20,000 pg/ml. Intra- and inter-assay variability was verified. Sample size was PND 28: *n* = 13; PND 48: *n* = 26; PND 71: *n* = 39.

After, circulatory T levels were assessed in young adult rats (PND 71) using an ELISA kit (Testosterone ELISA kit, Enzo Life Sciences, Cat. No. ADI-900-065). Thawing, punching, and mixing of Whatman card blood samples followed the same protocol as CORT assay. A total of 2.2 μL of SDR were added to 217.8 μl of each sample. Standard curve samples were prepared in duplicates as described by the manufacturer. Blood levels were assessed using a PowerWave TM XS2 Microplate spectrophotometer by comparing the sample's absorbance to a standard curve. The analytic range of the assay was 7.81–2,000 pg/ml. Sample size was BPA: *n* = 10; Hop: *n* = 9; BPA-Hop: *n* = 9: Control: *n* = 10.

### Behavioral Testing

Behavioral testing took place from PND 54 to 61 between 8:30 A.M. and 7 P.M. (EPM from 9 A.M. to 3:30 PM, SIT/SP from 8:30 A.M. to 7 P.M., and FST from 9 A.M. to 3 P.M.) Testing order was EPM, SIT, and FST. Animal groups and animals were counterbalanced within and between tested rat cohorts in order to minimize possible effects of time of day. Rats acclimated to the testing environment for at least 30 min prior to testing. Lighting was 200–400 lux in the animal vivarium and 800–900 lux (EPM, SIT) or 650–700 lux (FST) in the behavioral testing rooms. Behaviors were recorded using a video camera connected to a computer. Each apparatus was cleaned with a 70% ethanol solution between subjects.

#### Elevated Plus Maze

Animals were tested in the EPM on PND 54. The test consisted of a plus-shaped Plexiglas structure with two enclosed arms (H = 40 cm) and two open arms, all 50 × 10 cm, elevated 60 cm above the floor and separated from the experimenter's area (PC computer and overhead video camera). Rats were placed in the center of the EPM facing an open arm and were left to freely explore the apparatus for 5 min. Entry duration and frequency in open and closed arms (counted by entry of all four paws in an arm), ratio of time spent between the two zones [percentage of open arm time = (time in open arms/total time in arms) × 100], and head dips (rat dipping his head and shoulders over the open arms) were determined using ODLog. Increased open arm time and head dip frequency suggest reduced anxiety, whereas increased stretch-attend posture and open arm avoidance were indicative of elevated anxiety.

#### Social Interaction and Preference Test

On PND 55, social affiliation, social memory, and inclination/avoidance toward social novelty were assessed with the three-chamber SIT/SP paradigm (Kaidanovich-Beilin et al., 2011). In this paradigm, also known as Crawley's sociability and preference for social novelty protocol (2004), a rodent is presented with a choice between initiating direct approach with a novel conspecific rodent and spending more time in this chamber vs. spending time in the chamber containing no living congener (SIT). This setting is believed to allow testing the rat's desire to “socially” interact with a congener. In the second test (SP), the experimental rodent is presented with a choice between the now-familiar conspecific and a second novel conspecific. The apparatus consisted of a gray Plexiglas open arena (LWH: 75 × 75 × 30 cm) with an opaque gray floor and two similar wire cups used as empty or to contain the stranger individual. The arena was divided into three-chamber rectangular boxes (LW: 75 × 25 cm) with a removable door on each of them allowing access to the chamber and was kept on a table 90 cm above the floor. White curtains separated the test from the experimenter's recording area. The test was administered in three phases: habituation, social interaction, and social preference. Two stranger rats (S1 and S2) were used in this test and counterbalanced between subjects.

##### Habituation

Following placement in the middle chamber, the experimental rat was allowed to explore the two outer chambers containing identical empty wire cups for 5 min.

##### Social Interaction Test

During a first 10 min SIT session, the rat was presented with a choice between initiating direct interaction with a stranger rat (S1) under the wire cup in the first chamber or spending time with the empty cup (EC) in the second chamber. Time spent with S1 vs. the empty cup indicates the level of sociability.

##### Social Preference Test

In the second 10 min session (SP), performed immediately after the SIT, a novel conspecific (S2) was placed under the EC in the second chamber, and the experimental rat could freely interact directly or indirectly with the rats in either chamber. Stranger rat placement was counterbalanced between trials. After session completion, all animals were removed from the apparatus and returned to their home cage. Spending more time with S2 instead of S1 in the SP is an indicator of social novelty and social memory.

Measurements included time spent with S1 or EC in the SIT as well as time spent with S1 or S2 in the SP.

#### Forced Swim Test

On PND 60–61, the FST was used to assess behavioral despair and depressive-like behaviors. Briefly, rats were allowed to swim in transparent Plexiglas cylinders (*D* = 20.32 cm; *H* = 43.18 cm) filled with tap water (25 ± 1°C; *H* = 33 cm) for 15 min on the first exposure (pre-training) and for 5 min the next day (test). Movements pertaining to swimming/climbing and diving (i.e., active escape-like behaviors) and immobility (passive behavior, i.e., absence of other movements for ≥ 2.0 s except those needed to keep the head above water) were recorded. Subsequently, rats were dried and warmed by a heating pad in their home cages. Water was changed and cylinders cleaned between each rat.

### Statistical Analysis

Statistical analyses were performed using IBM SPSS^®^ Statistics v26. Data distribution and assumptions depending on each analysis were assessed for all variables. Outliers were identified by having a z score of 3 or more in comparison to the animal's group and were replaced by their closest extreme value ± 1. The appropriate transformation was performed when necessary. The *p*-level was set at 0.05 and data is presented as mean ± standard error of the mean (SEM) with effect sizes reported using partial eta squared. Time-dependent changes in CORT levels were analyzed using a one-way repeated measures ANOVA. Behavioral measures (i.e., EPM, SIT, and FST) and T levels were analyzed using a one-way independent groups ANOVA. When appropriate, Tukey HSD's *post-hoc* comparisons assessed between-group differences.

## Results

### Blood Serum Corticosterone Level

**Figure 2:**
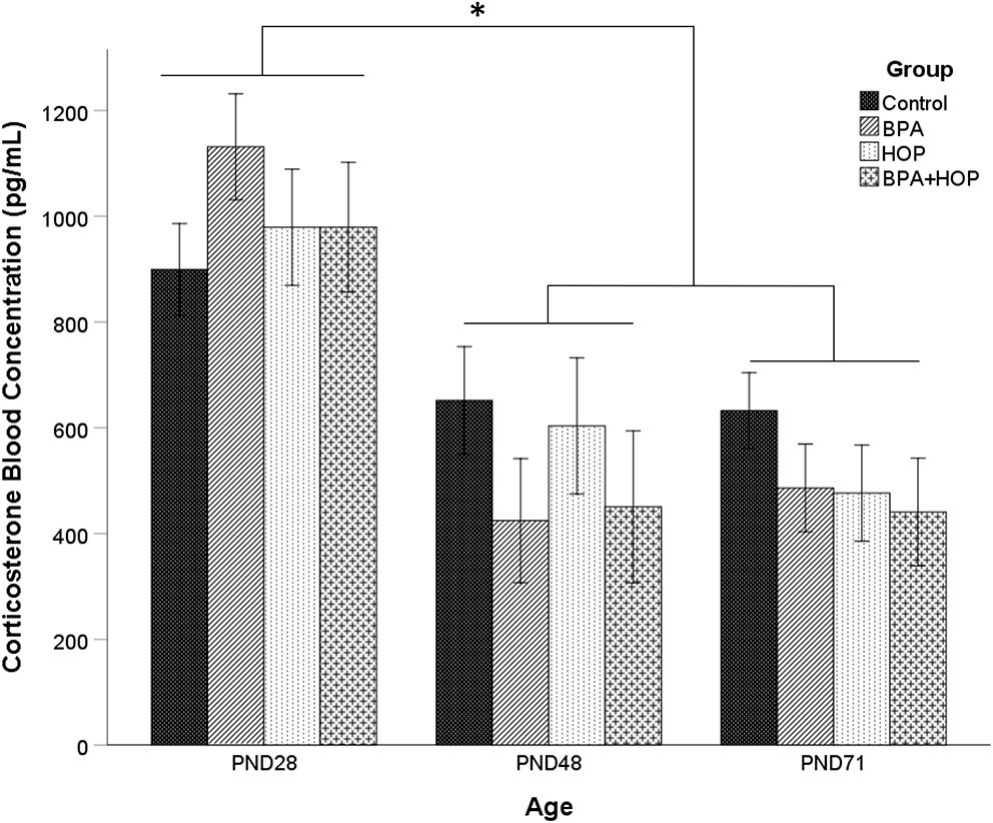
Blood samples were collected on the first (PND 28) and last day (PND 48) of gavage, and on the last day of experiment (PND 71) between 8 and 10 A.M. prior to gavage (Control: *n* = 8; BPA: *n* = 8; Hop: *n* = 6; BPA-Hop: *n* = 4). There was no significant effect of supplementation on corticosterone (CORT) levels, although a significant effect of time was found related to higher CORT levels in all the groups on PND 28 compared to the other collection intervals. Data are represented as mean ± SEM. *p < 0.05.

Variations of CORT concentrations in blood for all groups are presented in Figure 2. A one-way repeated measure ANOVA did not show an overall difference between groups, *F*_(3,19)_ =.42; *p* = 0.713; and no group X time interaction, *F*_(3,6)_ = 1.39; *p* = 0.245. However, there was a main effect of time [Wilks' Lambda = 0.28, *F*_(2,18)_ = 23.33, *p* < 0.001), with a decrease in CORT concentration after PND 28. *Post-hoc* analysis with a Bonferroni adjustment revealed that CORT levels on PND 28 (*M* = 997.31 pg/ml, *SD* = 52.8 pg/ml) were significantly higher than on PND 48 (*M* = 997.31 pg/ml, *SD* = 52.8 pg/ml) and PND 71 (*M* = 509.01 pg/ml, *SD* = 43.81 pg/ml). One outlier in the BPA group on PND71 was corrected. The overall elevated baseline levels suggest increased stress during the initial blood collection. However, a manufacturing defect in a portion of the Whatman paper prevented proper blood absorption onto the paper, leading samples to be excluded from the analysis. Due to missing values on PND 28, the mean imputation was used on those data to balance the sample size between time intervals. From the initial 10 rats per group, 8, 6, 5, and 4 were analyzed in the control, BPA, Hop, and BPA-Hop groups, respectively. The PND 28 interval served as a baseline measure of CORT secretion collected prior to initiation of the dietary supplementation. At this interval, all rats had been exposed to the same diet and vivarium/housing conditions.

### Blood Serum Testosterone Level

**Figure 3:**
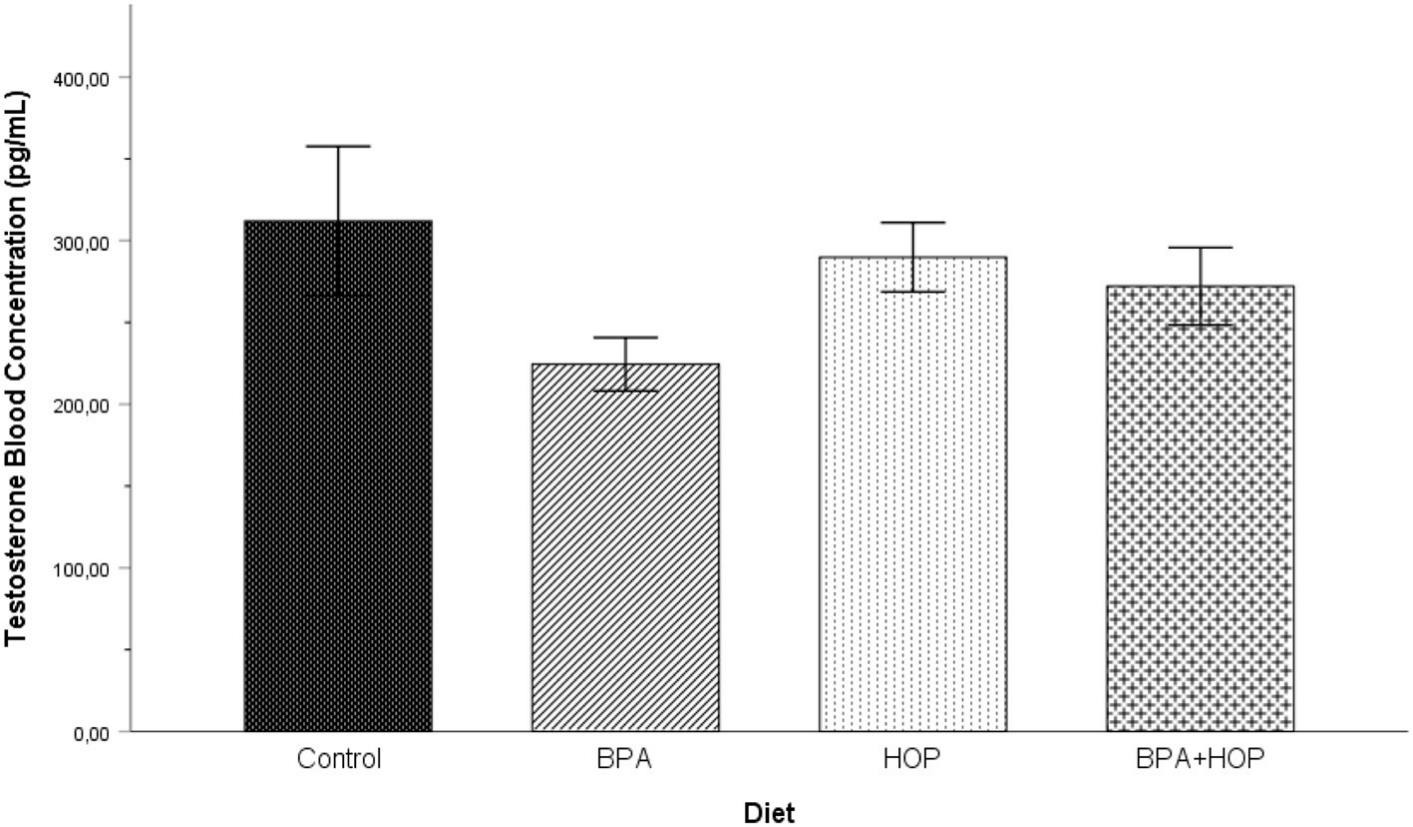
Blood testosterone (T) levels were assessed on PND 71 (Control: *n* = 10; BPA: *n* = 10; Hop: *n* = 9; BPA-Hop: *n* = 9). No significant changes were detected between groups. Findings support a trend toward decreased T levels in BPA-treated rats. Data are represented as mean ± SEM.

Testosterone concentrations measured on PND 71 are shown in Figure 3. In order to respect normal distribution, log transformation was applied on the T levels data. One outlier in the Hop group was corrected. A one-way ANOVA was conducted on the transformed data to assess whether exposure to BPA, hop extracts, or the combination of those had long-lasting effects on the T levels concentration in adulthood. The analysis failed to reveal any significant group differences, *F*_(3,37)_ = 1.88; *p* = 0.150; η^2^ = 0.14.

### Behavioral Testing

#### Elevated Plus Maze

**Figure 4:**
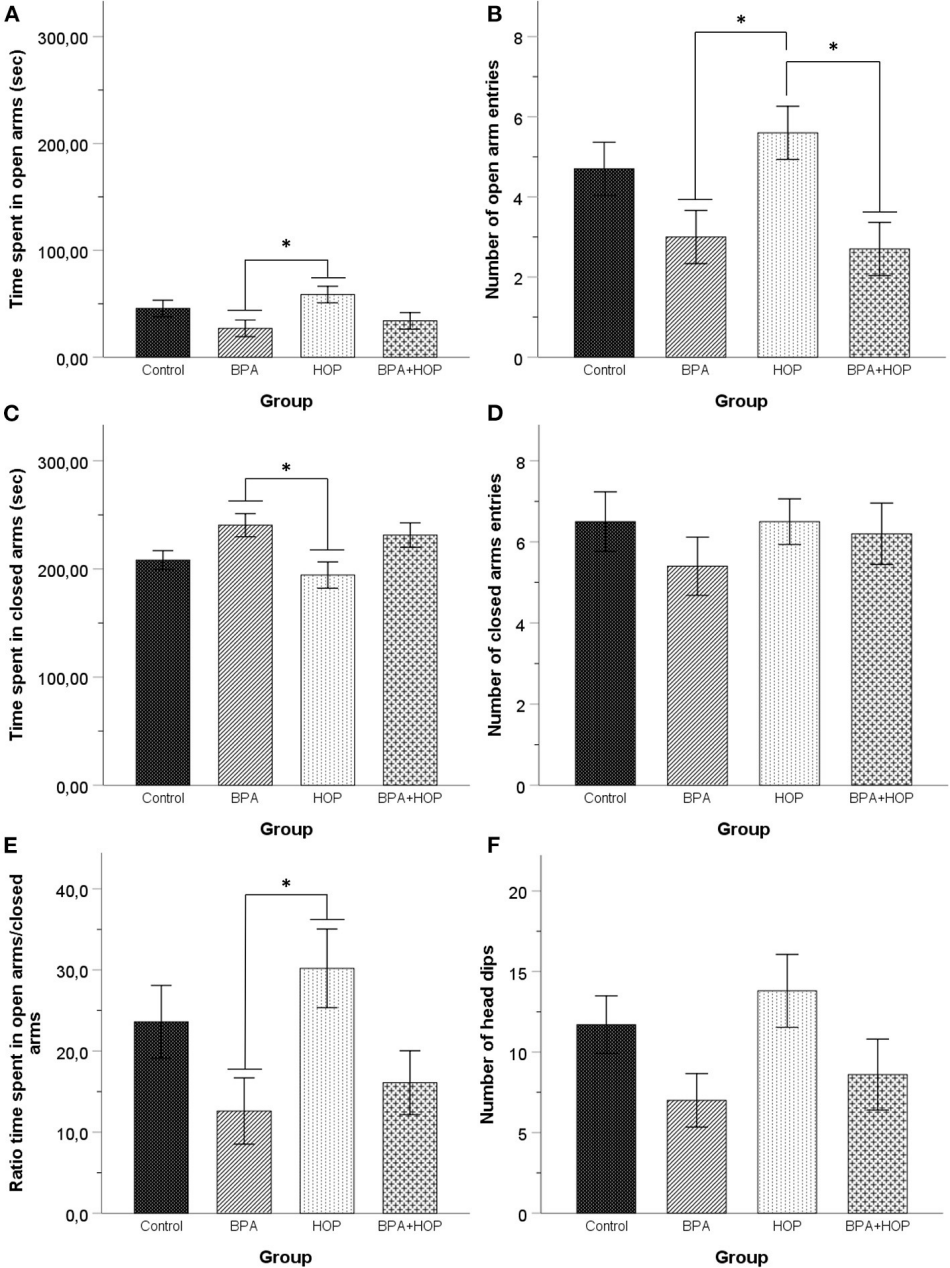
All rats were tested in the elevated plus maze on PND 54 (Control: *n* = 10; BPA: *n* = 10; Hop: *n* = 11; BPA-Hop: *n* = 10). Rats in the BPA and the Hop groups differed significantly in **(A)** time spent in open arms; **(B)** number of open arm entries; **(C)** time spent in closed arms. However, **(D)** they did not differ in the number of closed arm entries. **(E)** The ratio of time spent in the open over closed arms was calculated using the ratio of time spent in closed arms over the total amount of time recorded in both open and closed arms. BPA-treated rats spent a significantly smaller time ratio in the open arms compared to the Hop group. **(F)** There was no between-group differences in the number of head dips. Data are represented as mean ± SEM. *p < 0.05.

Performance in the EPM is presented in Figure 4. One-way ANOVA revealed a significant difference between groups in time spent in open arms, *F*_(3,36)_ = 3.19; *p* = 0.035; η^2^ = 0.21, and the number of entries in open arms, *F*_(3,36)_ = 4.34; *p* = 0.01; η^2^ = 0.27]. Tukey HSD's *post-hoc* comparisons indicated that the Hop group (58.61 ± 29.03 s) spent more time in the open arms than the BPA group (26.98 ± 23.49 s, *p* = 0.032) while hop animals (5.6 ± 2.37) made more open arm entries than the BPA (3 ± 2.36, *p* = 0.042) and BPA-Hop (2.7 ± 1.57, *p* = 0.019) groups.

One-way ANOVA determined between-group difference for time spent in closed arms, *F*_(3,36)_ = 3.85; *p* = 0.017; η^2^ = 0.24, but not for the number of closed arm entries, *F*_(3,36)_ = 4.34; *p* = 0.01; η^2^ = 0.27. We calculated the ratio of time spent in open vs. closed arms. Results were compared using a one-way ANOVA and showed a significant effect of group on the ratio of time spent in open vs. closed arms, *F*_(3,36)_ = 3.2; *p* = 0.035; η^2^ = 0.21. Tukey HSD's *post-hoc* test again supported reduced time in the closed arms (*p* = 0.022) and a significantly higher ratio of time spent in open arms/closed arms (*p* = 0.036) in Hop (194.32 ± 38.44 s and 30.07 ± 4.88, respectively), compared to BPA (240.54 ± 33.70 s and 12.61 ± 4.06, respectively) animals. One outlier in the Hop group was corrected. A significant group difference was observed in head dips duration, *F*_(3,36)_ = 3.54; *p* = 0.024; η^2^ = 0.23, but not frequency, *F*_(3,36)_ = 2.34; *p* = 0.089; η^2^ = 0.16. Tukey HSD's *post-hoc* comparisons revealed that hop rats (38.09 ± 19.60 s) spend more time performing this behavior than BPA-treated rats (15.84 ± 15.99 s, *p* = 0.028). Together, these results support increased anxiety in BPA exposed rats compared to hop extracts-treated rats.

#### Social Interaction and Preference Test

**Figure 5:**
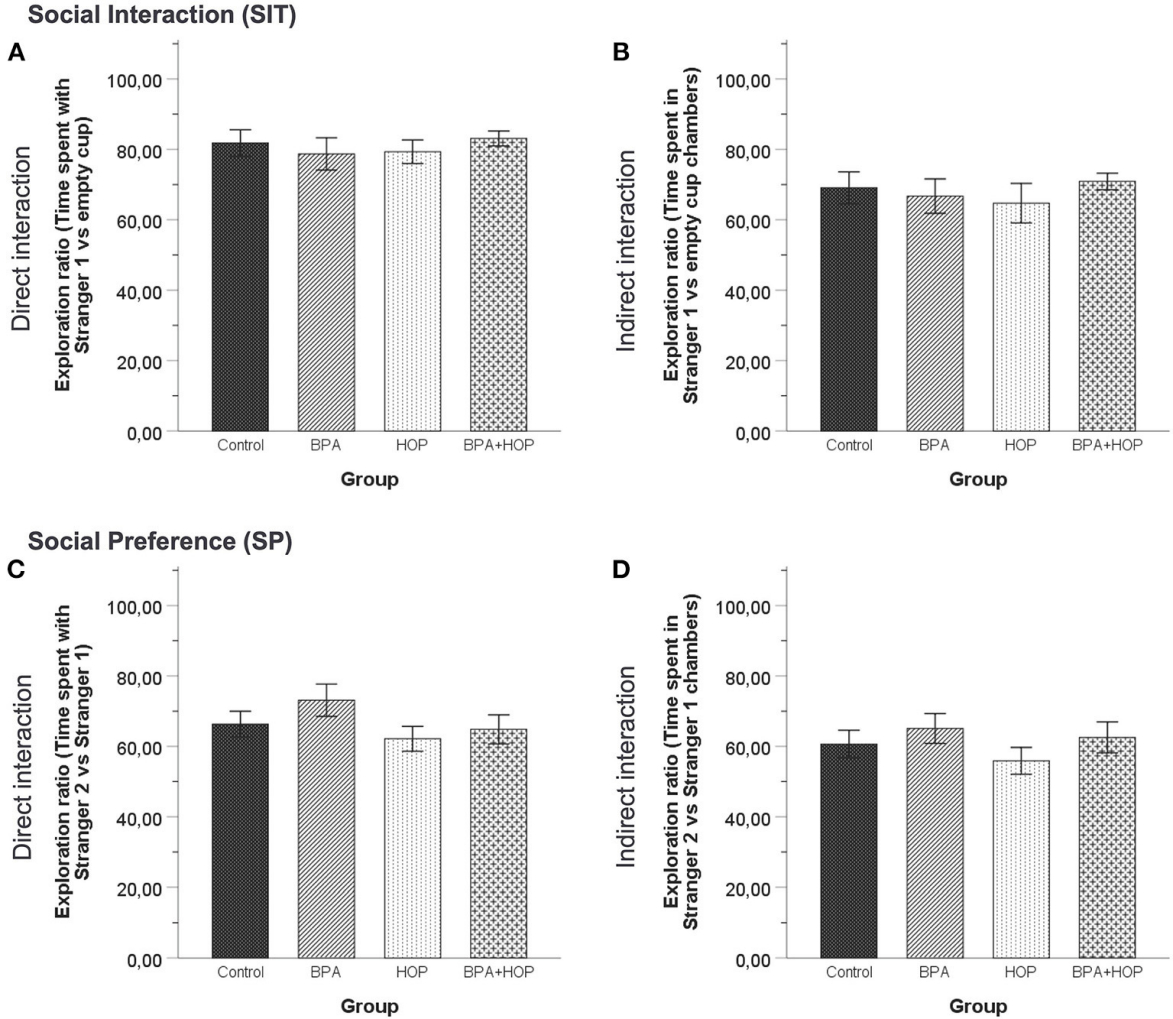
All rats were tested in the social interaction test on PND 55 (Control: *n* = 10; BPA: *n* = 10; Hop: *n* = 11; BPA-Hop: *n* = 10). No significant differences in sociability as measured via the time spent directly interacting with the stranger rat vs. the empty cup **(A)** or indirectly interacting by being in the stranger rat's chamber vs. the empty chamber **(B)** were noted. Social preference/recognition was also not altered, as measured via the time spent directly interacting with stranger rat 1 vs. stranger rat 2 **(C)** or indirectly interacting by being in the stranger rat 1's chamber vs. stranger rat 2's chamber **(D)**. Data are represented as mean ± SEM.

Behavioral activities in both the SIT and SP are reported in Figure 5. One-way independent groups ANOVA did not reveal any difference between groups in the SIT regarding the time spent interacting with the S1, *F*_(3,36)_ = 1.395; *p* = 0.26, or the time spent interacting with the EC, *F*_(3,36)_ = 0.162; *p* = 0.921. In the SP, there was no difference determined by analysis regarding time spent interacting with the S1, *F*_(3,36)_ = 1.52; *p* = 0.225, and with the S2, *F*_(3,36)_ = 1.77; *p* = 0.169. Those results underline a similar level of social behavior between groups, with a trend for the Hop group to show increased exploratory and social behavior in both tests.

#### Forced Swim Test

**Figure 6:**
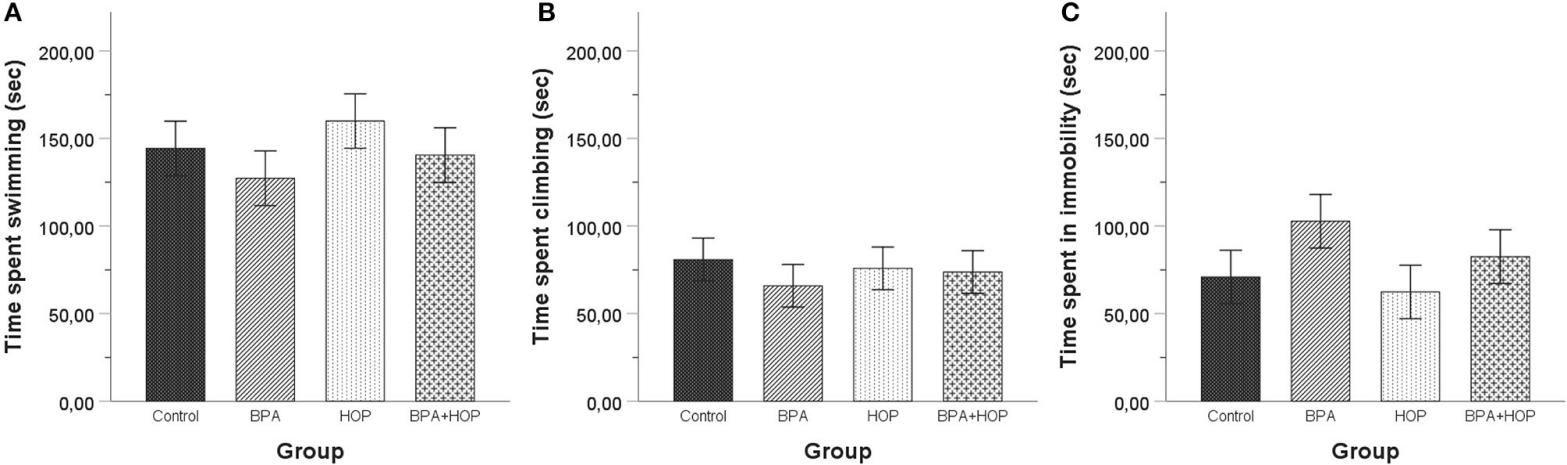
All rats were tested in the forced swim test on PND 60 (habituation) and PND 61 (5-min swim test). No differences were observed between the rats in time spent swimming **(A)**, climbing **(B)**, or being immobile **(C)**. A trend toward spending less time swimming and climbing and more time motionless was apparent in BPA-treated compared to control and Hop-treated rats. Data are represented as mean ± SEM.

Behavioral responses of the groups are reported in Figure 6. No between-group differences were found using one-way ANOVA of time spent swimming, *F*_(3,36)_ = 0.745; *p* = 0.532; η^2^ = 0.058, climbing, *F*_(3,36)_ = 0.260; *p* = 0.854; η^2^ = 0.021, or being immobile, *F*_(3,36)_ = 1.305; *p* = 0.288; η^2^ = 0.098.

## Discussion

To our knowledge, this study is the first to examine the effects of adolescent exposure to hop extracts on adulthood endocrine and emotional responses, and to evaluate the interplay between hops and BPA exposure on corticosterone, testosterone, and socioemotional behaviors. The paucity of research addressing adolescence-circumscribed BPA administration (Diaz Weinstein et al., 2013; Bowman et al., 2014; Gao et al., 2020) contrasts numerous studies on the effects of gestational, perinatal, and peripubertal exposure to BPA (Poimenova et al., 2010; Matsuda et al., 2012; Patisaul et al., 2012; Ogi et al., 2015; Rebuli et al., 2015; Hong et al., 2016). Our findings lend further support to the idea of the adolescent period having an important role in differentiating developmental sensitivity to BPA's impact on adulthood testosterone secretion, sociability, and anxiety-like behavior in male rats.

### Anxiety-, Depressive-Like Behavior, and Basal CORT Secretion

Previous studies have supported gestational and perinatal BPA exposure to exert lasting anxiogenic effects in rodents (Xu et al., 2012; Chen et al., 2015), while others have supported reduced anxiety-like behaviors (Farabollini et al., 1999), demasculinization of male behavior (Jones and Watson, 2012), or no effects (Wise et al., 2019). During adolescence, daily BPA dietary exposure failed to significantly affect EPM open arm time and entries in adult rats. Our findings are consistent with recent observations that also failed to show alterations of anxiety levels measured in the EPM in adult male rats that had been orally exposed daily to a 1 mg phthalates/kg mixture as adolescents (PND 27–50), supporting no significant impact of adolescent phtalates exposure on anxiety-like behavior in male rats (Sellinger et al., 2020). Notably, however, adolescent exposure to BPA inhibited hops-induced open arm entries in co-exposed rats. This co-exposure effect in regulating behavioral response substantiates a number of studies that have reported the ability of various dietary/naturally occurring compounds to regulate different BPA genomic and biochemical effects upon co-exposure at physiological and pharmacological dose regimens (Sonavane and Gassman, 2019).

Interestingly, the behavioral trends observed in the EPM are concordant with BPA-induced anxiety reported at other developmental stages (Matsuda et al., 2012; Patisaul et al., 2012; Diaz Weinstein et al., 2013; Xu et al., 2015). As for hops, anxiolytic effects have been observed using elevated concentrations (250–1,000 mg/kg) (Negri et al., 2010) with lower hops or xanthohumol dosages (2.5, 5, 10, and 20 mg/kg) having no significant effect (Zanoli et al., 2005; Ceremuga et al., 2013). Although mechanisms remain to be determined, the absence of xanthohumol's effects in the EPM (Ceremuga et al., 2013) suggests a minor role of this chalconoid in mediating hops' anxiolytic actions. Rather, Zanoli and Zavatti (2008) showed hop α and β acid fractions to contribute to anxiolytic effects, the former playing a major role in hops-induced pentobarbital effects. The elevated α bitter acids concentration of our extract (see Table 1) thus likely promoted open arm exploration. At present, controlled pharmacokinetic studies addressing the distinctive roles of the various prenylflavonoids and bitter acids in hop extracts are lacking and future research should be encouraged.

Hops and BPA supplementation had no immediate or delayed impact on basal CORT secretion measured at PND 28, 48, and 71. A higher CORT secretion was observed in all rats (independent of treatment) on PND 28, likely due to the novelty effect of initial blood sampling. Corticosterone then stabilized upon subsequent PND 48 and 71 measurements. This is consistent with human studies reporting no differences in circulatory cortisol concentrations in both male and female adults exposed to hop extracts (two 0.2 g capsules; Kyrou et al., 2017). Conversely, recent research supports state-dependent regulatory effects of hop extracts on CORT secretion. In fact, Donoso et al. (2020) recently demonstrated that an 8-week xanthohumol (0.015%; 10 mg/kg/day) supplementation period in adult rats exposed to early life stress (3 h daily maternal separation from PND 2–12) reduced basal and stress-induced CORT secretion and reduced anxiety-like behaviors observed in non-supplemented rats. As for BPA, gestational and lactational supplementation periods have been associated with elevated basal CORT levels in adolescent female, but not male, rats while levels in both sexes saw increases following a mildly stressful Y-maze experience (Poimenova et al., 2010). Therefore, it is possible that the trend toward reduced open arm entries observed in the EPM in BPA-treated male rats in this study could be associated with increased CORT secretion induced by testing, although this was not measured. Future studies will benefit from comparing effects in male and female rodents and further exploring HPA axis regulation under stressful conditions.

Furthermore, Donoso's et al. study (2020) reported adulthood xanthohumol supplementation to attenuate depressive-like behaviors in the FST after early life stress exposure in rats. In our study, long-term dietary supplementation with hops or BPA, performed in the absence of concomitant exposure to other stressors, failed to affect behavior in the FST. This is consistent with Zanoli's et al. (2005) findings demonstrating no effects following acute administration of 2.5 and 20 mg/kg of hop extracts at three timepoints (24, 5, and 1 h) prior to FST exposure, whereas doses of 5 and 10 mg/kg resulted in decreased immobility. Our findings further support a complex dose-response profile. To our knowledge, this study is the first to examine the effects of adolescent hops supplementation and co-exposure with BPA on depressive-like behavior. Previous studies have reported enhanced depressive-like behaviors in rodents exposed to low-dose BPA pre- or perinatally (via oral gavage or lactation) (Fujimoto et al., 2013; Chen et al., 2014), suggesting developmental stage-specific effects of BPA. Proposed mechanisms for BPA modulation of depressive-like behaviors have been related to changes in GABA_(A)α2_ and Erβ (Xu et al., 2015), glutamate NMDA and AMPA receptor subunit GluR1 expression (Xu et al., 2012), and sex-specific reduced serotonin levels in males (Xin et al., 2018).

### Adulthood Sociability, Social Recognition, and Testosterone Secretion

Consistent with the Hicks et al. (2016) study on post-natal BPA supplementation (2 mg/L), our findings indicated no significant change in adult sociability associated with adolescent BPA exposure. Similarly, although at much lower doses, prenatal and lactational BPA doses of 40 and 400 μg/kg/day in male Sprague–Dawley rats failed to affect adult play behavior (Dessì-Fulgheri et al., 2002). Moreover, using similar dosages, perinatal exposure in Long Evans rats failed to show lasting impact on adult social memory (Wise et al., 2019). Notably, using transgenerational and prenatal BPA administration regimens, Wolstenholme et al. (2019) and Rebuli et al. (2016) demonstrated earlier periods of development to be more sensitive to lasting effects on sociability. Furthermore, a recent study by Gao et al. (2020) supports non-linear dose-related effects of an 18-day BPA supplementation paradigm in adolescent male mice (PND 28–46) on pubertal social play and adulthood social interaction. For instance, of three tested BPA doses, only the lowest (0.04 mg/kg/day) affected pubertal social play. None of the studied doses affected social investigation, as measured by anogenital sniffing time. When tested as adults in the SIT, BPA reduced sociability [i.e., time mice spent in direct contact with the stranger mouse (vs an empty cylinder)], but only the middle 0.4 mg BPA dose affected social recognition (i.e., reduced interaction with the stranger vs. the familiar mouse). Adult sexual behavior with females was unaltered and only the 4 mg/kg/day reduced testosterone levels. Taken with our results using a higher dosage, these findings indicate complex impacts of adolescent BPA exposure, marked by non-linear dose-response profiles. To our knowledge, this study is the first to assess the effects of adolescent hop extracts on social behavior. Here, we demonstrate that such supplementation, alone or in conjunction with BPA, did not lead to long-term alterations on sociability and/or social memory. We therefore suggest future research to investigate longer exposure periods that would include the perinatal window.

Testosterone plays an important role in certain facets of social behavior, such as motivating male sexual behaviors (Karlsson et al., 2015). In this context, BPA (4 mg/kg) has recently been associated with reduced androgen receptor expression and serum/brain T levels, coincident with increased male-male sociosexual interactions at the expense of male-female interactions (Gao et al., 2020). Using a higher dosage, our findings do not support adolescent BPA-hops supplementation to influence adulthood T levels. After puberty, reduced T levels have been noted in previous studies using lower (20 mg/kg), equal (40 mg/kg), or higher (200 mg/kg) BPA dosages (Della Seta et al., 2006; Nakamura et al., 2010). These changes appear to be enacted in a dose-response manner (Ullah et al., 2018). Nonetheless, consensus is not achieved as other studies failed to show effects, even using long-term BPA exposure at similar (i.e., 50 mg/kg) dosages (Jašarević et al., 2011). Hops supplementation during adolescence had no impact on T secretion. This contrasts Kazeruni's et al. (2014) study showing upregulation of T in mice supplemented with pharmacological doses of hops (50–150 mg/kg) as adults.

## Conclusion

Many environmental pollutants and endocrine disruptors exert important and lasting negative health effects. To this day, the level of vulnerability to pollutants within specific developmental time windows remains unclear. One impactful way to countermeasure adverse effects has been through incorporation of nutraceuticals in the diet. In this study, we determined that BPA- and hop-exposed rats differed in anxiety, with hops-exposed animals showing reduced anxiety. Under normal conditions, CORT secretion was unaffected by BPA or hops supplementation, not excluding that supplementation could alter HPA reactivity following stressor exposure. Our findings suggest that BPA administration during adolescence has no significant delayed effects on depressive-like behaviors and sociability. Our study is the first to characterize co-exposure effects of BPA and hops on behavioral responses. Similar to other studies assessing co-exposure, we selected a pharmacological BPA dosage (40–200 mg/kg: Dolinoy et al., 2007; Wu et al., 2013; Wahby et al., 2017; Saadeldin et al., 2018). Future studies assessing lower doses are necessary to establish ecological exposure profiles. Notably, different studies support estrogenic actions of BPA and hop extracts and that BPA can disrupt estrogen receptors in the developing brain. In this context, sex differences certainly need to be established. To our knowledge, only one study has assessed differential effects of pubertal exposure to BPA upon reaching adulthood. Yu et al. (2011) indeed observed a BPA-induced masculinization of female social behavior, while male behavior was not altered. Combined with other studies, our findings support anxiety to be particularly sensitive to BPA exposure during critical maturing stages. Thus, our findings further support mutual influences between environmental polyphenolic compounds such as hops and endocrine disruptors.

## Data Availability Statement

The raw data supporting the conclusions of this article will be made available by the authors, without undue reservation.

## Ethics Statement

All procedures in this study were carried out in accordance with the Canadian Council on Animal Care and were approved by the University of Ottawa Animal Care Committee.

## Author Contributions

AM, LVdB, and EP performed data acquisition, analysis, and interpretation. HP contributed to the project's conception and results interpretation. All authors participated in the writing of the manuscript and approved the final version.

## Conflict of Interest

The authors declare that the research was conducted in the absence of any commercial or financial relationships that could be construed as a potential conflict of interest.
